# Comprehensive analysis of the codon usage patterns in the polyprotein coding sequences of the honeybee viruses

**DOI:** 10.3389/fvets.2025.1567209

**Published:** 2025-07-04

**Authors:** Yeşim Aktürk Dizman

**Affiliations:** Department of Biology, Faculty of Arts and Sciences, Recep Tayyip Erdoğan University, Rize, Türkiye

**Keywords:** honeybee viruses, polyprotein, codon usage bias, natural selection, host adaptation

## Abstract

Honeybee viruses (HVs) are some of the most significant pathogens affecting these insects and are commonly found in beehives across the globe. This viral infection leads to substantial economic losses in the beekeeping industry. To understand the evolution and adaptation of HVs, such as Acute Bee Paralysis Virus (ABPV), Kashmir Bee Virus (KBV), Chronic Bee Paralysis Virus (CBPV), and Sacbrood Virus (SBV), a detailed analysis of codon usage bias (CUB) was conducted, as no prior studies on this topic had been reported. Analysis of nucleotide content and RSCU revealed that the polyprotein coding sequences of the four HVs were rich in A/U nucleotides, with the third base of synonymous codons predominantly A/U. The polyprotein coding sequences showed a higher effective number of codons (ENC) value, suggesting lower CUB. The ENC plot, PR2 plot, and neutrality analyses indicated that natural selection predominantly shapes the codon usage pattern of polyprotein coding sequences, with minimal influence from mutation pressure. Analyses of the codon adaptation index (CAI) and relative codon deoptimization index (RCDI) showed a strong relationship between HVs and their hosts. These findings could offer essential insights into the overall codon usage patterns of HVs and help in understanding the mechanisms that influence codon usage and genetic evolution in HVs.

## 1 Introduction

Honeybees are vital pollinators that underpin global biodiversity and agricultural productivity (1, 2). However, over recent decades, honeybee populations have been declining at an alarming rate due to various factors, including climate change, pesticide exposure, habitat loss, and the spread of infectious pathogens (3–5). Among these, honeybee viruses such as Deformed wing virus (DWV), Sacbrood virus (SBV), Black queen cell virus (BQCV), Kashmir bee virus (KBV), Acute bee paralysis virus (ABPV), Israeli acute paralysis virus (IAPV) and Chronic bee paralysis virus (CBPV) play a key role in the health deterioration of honeybee colonies (6–8). SBV and DWV are classified under the genus *Iflavirus* in the family *Iflaviridae*. In contrast, KBV, ABPV, and IAPV belong to the genus *Aparavirus* within the family *Dicistroviridae*, while BQCV is part of the genus *Triatovirus*, also in the family *Dicistroviridae* (9–11). The CBPV remains unclassified (12). These viruses, often vectored by ectoparasitic mites like Var*roa destructor*, contribute significantly to colony collapse disorder, posing a severe threat to ecosystem stability and agricultural sustainability (5, 13). Understanding the molecular mechanisms driving the evolution and adaptation of these viruses to their hosts is critical for developing effective strategies to safeguard honeybee populations.

The genetic code is made up of 64 standard codons, with UGA, UAG, and UAA serving as stop codons that signal the end of translation. The remaining 61 codons code for 20 standard amino acids. Of these, tryptophan and methionine are each represented by a single codon, UGG and AUG, respectively, while the other 18 amino acids are coded by two or more codons. The genetic code’s degeneracy enables multiple codons, known as synonymous codons, to encode the same amino acid (14, 15). However, these synonymous codons are not utilized with equal frequency, leading to codon usage bias (CUB) (16). CUB is shaped by a variety of factors, including mutational pressure, natural selection, gene length, GC content, and tRNA availability (17, 18). Viral genomes exhibit unique features compared to those of eukaryotes and prokaryotes, including their dependence on hosts for replication, protein synthesis, and transmission. This interaction between viruses and their hosts is believed to play a crucial role in viral survival, adaptation, evasion of the host’s immune system, and evolution (19–21). Thus, understanding codon usage in viruses offers insights into molecular evolution, enhances our knowledge of viral gene expression regulation, and supports vaccine development by optimizing the effective expression of viral proteins needed to elicit immunity.

CUB is increasingly recognized as an important tool for understanding virus-host interactions (22, 23). Viruses with codon usage patterns that align closely with those of their hosts may achieve more efficient replication and protein expression, reflecting a process of host adaptation. Conversely, deviations in codon usage may indicate evolutionary constraints or shifts in host-virus dynamics (24, 25). Although CUB has been extensively studied in many genomes (26, 27), research on honeybee viruses remains relatively limited (28–30). Hence, this study seeks to fill this gap by conducting a comparative analysis of codon usage bias in HVs, including ABPV, CBPV, KBV, and SBV, which could ultimately contribute to the development of targeted strategies to manage honeybee health, mitigate the impact of viral pathogens on pollinator populations, and provide a foundation for future research on virus evolution and host adaptation.

## 2 Materials and methods

### 2.1 Retrieving coding sequences data

CUB analysis was performed on ABPV, CBPV, KBV, and SBV. Since the codon usage patterns of BQCV, DWV, and IAPV have been previously studied (29), they were not included in the present study. The complete nucleotide sequences of the polyprotein coding sequences from 43 ABPV, 57 CBPV, 8 KBV, and 96 SBV were obtained in FASTA format from the National Center for Biotechnology Information (NCBI).^1^ The accession numbers are provided in Supplementary Table 1.

### 2.2 Codon usage analysis

The total nucleotide content percentages (U, A, C, and G), nucleotide content at the 3rd position of synonymous codons (U3s, A3s, C3s, and G3s %), as well as the overall GC and AU content percentages, along with their frequencies at the first (GC1%), second (GC2%), and third positions (GC3s %) of synonymous codons, were determined with the CodonW 1.4.2 (31), which provides both menu-driven and command-line interfaces to facilitate flexible data analysis.

The effective number of codons (ENC) index measures the extent of bias in synonymous codon usage. The ENC values were computed with CodonW 1.4.2 (31). ENC values range between 20 and 61, with values below 35 suggesting a CUB; where lower values reflect a stronger bias.

### 2.3 Relative synonymous codon usage analysis

To evaluate synonymous codon usage independently of amino acid composition across various gene samples, the relative synonymous codon usage (RSCU) values for each codon in every sequence were computed using CodonW 1.4.2 (31). An RSCU value above 1.0 demonstrates a codon that is used more frequently (high bias), while a value below 1.0 signifies a codon that is used less frequently (low bias). An RSCU value of 1.0 signifies an absence of bias, indicating equal preference among codons encoding a particular amino acid (32). An RSCU value exceeding 1.6 indicates an overrepresented synonymous codon, whereas a value below 0.6 signifies an underrepresented synonymous codon (33).

### 2.4 Relative dinucleotide abundance analysis

Relative dinucleotide abundance analysis focuses on analyzing the frequency patterns of dinucleotide pairs within a specific sequence. This analysis offers an understanding of the compositional biases and possible functional roles of dinucleotide patterns in nucleic acid sequences (34). The formula for calculating dinucleotide frequency, as described by Kariin and Burge (35), is ρxy = ƒxy/ƒxƒy. Here, ƒx and ƒy are the frequencies of the individual nucleotides X and Y, ƒxy represents the observed frequency of the dinucleotide XY, and ρxy denotes the relative frequency of the dinucleotide XY. Dinucleotides with ρxy values exceeding 1.23 were categorized as overrepresented, whereas those below 0.78 were considered underrepresented. The compseq software was used to carry out this analysis,^2^ as it calculates the composition of sequence motifs of a specified length (e.g., dimers, trimers) within the input sequences.

### 2.5 ENC plot analysis

An ENC plot analysis was carried out to explore the factors affecting CUB in the polyprotein coding sequences of HVs. The ENC plot illustrates the correlation between ENC and GC3 values. If the observed ENC values are close to or align with the expected ENC curve, it indicates that mutation pressure is the main factor shaping codon usage. However, deviations below the expected curve imply that other factors, such as natural selection, are also influencing the CUB (36). The formula used to compute the expected ENC values was as follows (37):

$$\mathrm{EN}C_{\exp}=2+\mathrm{GC}3s+(\frac{29}{\mathrm{GC}3s^{2}+{\left(1-\mathrm{GC}3s\right)}^{2}})$$

### 2.6 Neutrality plot analysis

A neutrality plot is commonly used to identify whether mutational forces or evolutionary forces predominantly influence the CUB of a gene. The plot was generated with GC12 displayed on the y-axis and GC3 plotted on the x-axis. In this graph, a regression line slope near 0 indicates the absence of mutation pressure (with natural selection playing a dominant role), while a slope close to 1 signifies complete neutrality (with mutation pressure being dominant) (38).

### 2.7 Parity rule 2 analysis

Parity rule 2 (PR2) analysis was applied to evaluate the relative roles of natural selection and mutation pressure on the CUB in the polyprotein coding sequences of HVs. In the PR2 plot, the x-axis displays the value of GC bias at the 3rd codon position [G3/ (G3 + C3)], while the y-axis shows the value of AU bias at the 3rd codon position [A3/ (A3 + U3)]. The origin point (0.5, 0.5) signifies an equal balance between A and T (A = T) as well as between G and C (G = C) (39). Departures from the origin point imply the effects of mutation pressure and natural selection on codon usage.

### 2.8 Codon adaptation index and relative codon deoptimization index analysis

The codon adaptation index (CAI) was determined using the CAI calculation tool provided by the CAIcal server (40), which performs various computations related to codon usage and the adaptation of DNA or RNA sequences to host organisms. CAI analysis is used to estimate gene expression levels and assess how effectively viral genes have adapted to their host organisms by comparing them with the reference host’s RSCU. Higher CAI values indicate higher levels of gene expression and greater codon bias; the values range from 0 to 1.

Relative codon deoptimization index (RCDI) analysis was conducted utilizing the RCDI server (41). An RCDI value of 1 signifies that the virus has a codon usage pattern well-adapted to the host. In contrast, a value greater than 1 indicates reduced adaptation (42). An elevated RCDI value signifies a larger deviation from the codon usage pattern of host. The host codon usage was extracted from the codon and codon pair usage tables (CoCoPUTs) (43).

### 2.9 Correspondence analysis

Correspondence analysis (COA), a multivariate statistical analysis technique, is commonly employed to explore the connections among samples and variables (44). COA was conducted using the CodonW 1.4.2 (31) with RSCU values for individual codons to investigate the codon usage patterns of the polyprotein coding sequences in ABPV, CBPV, KBV, and SBV. Each polyprotein coding sequences was depicted as a 59-dimensional vector space, representing 59 synonymous codons, excluding TAA, TAG, TGA, ATG, and TGG with each point corresponding to the RSCU values of the synonymous codons. The first two axes were sufficient to explain a larger portion of the data compared to the other axes, so codons were plotted along these two axes.

### 2.10 General average hydropathicity and aromaticity

The GRAVY index is determined by averaging the hydropathy values of each amino acid. Its score spans from −2 to 2, with a positive value indicating a hydrophobic protein and a negative value indicating a hydrophilic protein (42). AROMA values indicate whether the gene products contain the aromatic amino acids (Tyr, Phe, and Trp) that the codons encode (45). These two indices are employed to examine the influence of natural selection on the codon usage pattern (33).

### 2.11 Correlation analysis

Correlation analysis was conducted to characterize the relationships between nucleotide contents and the codon usage patterns of the polyprotein coding sequences in ABPV, CBPV, KBV, and SBV. Correlation analyses were conducted using Pearson correlation method with OriginPro 9.0 software, and statistical significance was considered at *p* < 0.05.

## 3 Results

### 3.1 Nucleotide content in the polyprotein coding sequences

In the polyprotein coding sequences of the four HVs, nucleotide A had the highest mean composition at 29.68%, followed by U at 27.46%, C at 21.77%, and G at 21.09% (Table 1). At the 3rd positions of synonymous codons, the nucleotide composition displayed a distinct pattern, with U3s being the most prevalent at 40.01%, followed by A3s at 39.49%, C3s at 22.97%, and G3s at 20.83%. This indicates an enrichment of A/U-ending codons in the polyprotein coding sequences. The average contents of AU and GC were 57.14 and 43.02%, respectively. Furthermore, the GC content varied across different codon positions, with GC1 being the highest at 47.86%, followed by GC12 at 45.31%, GC2 at 42.76%, and GC3s at 34.94%.

**Table 1:** Codon usage indices of polyprotein coding sequences in four HVs.

Items	ABPV	CBPV	KBV	SBV	MEAN
A%	34.47	21.40	32.99	29.86	29.68
U%	29.14	25.58	25.77	26.10	26.65
G%	19.29	20.85	19.72	21.77	20.41
C%	17.11	32.17	21.52	14.48	21.32
GC	0.365	0.536	0.410	0.410	0.430
U3s	0.458	0.297	0.382	0.464	0.400
C3s	0.143	0.419	0.236	0.121	0.230
A3s	0.493	0.242	0.466	0.379	0.395
G3s	0.157	0.220	0.159	0.297	0.208
GC3s	0.231	0.539	0.315	0.313	0.349
ENC	44.10	54.10	51.86	49.05	49.78
AU	63.61	46.98	58.75	59.22	57.14
AU3	73.88	42.73	65.50	66.06	62.04
GC1	42.92	53.03	48.05	47.43	47.86
GC2	40.15	48.74	41.19	40.98	42.76
GC12	41.53	50.89	44.62	44.20	45.31
GRAVY	−0.294	0.271	−0.265	−0.251	−0.135
AROMA	0.097	0.078	0.100	0.098	0.093

### 3.2 Codon usage bias of the polyprotein coding sequences

**Figure 1:**
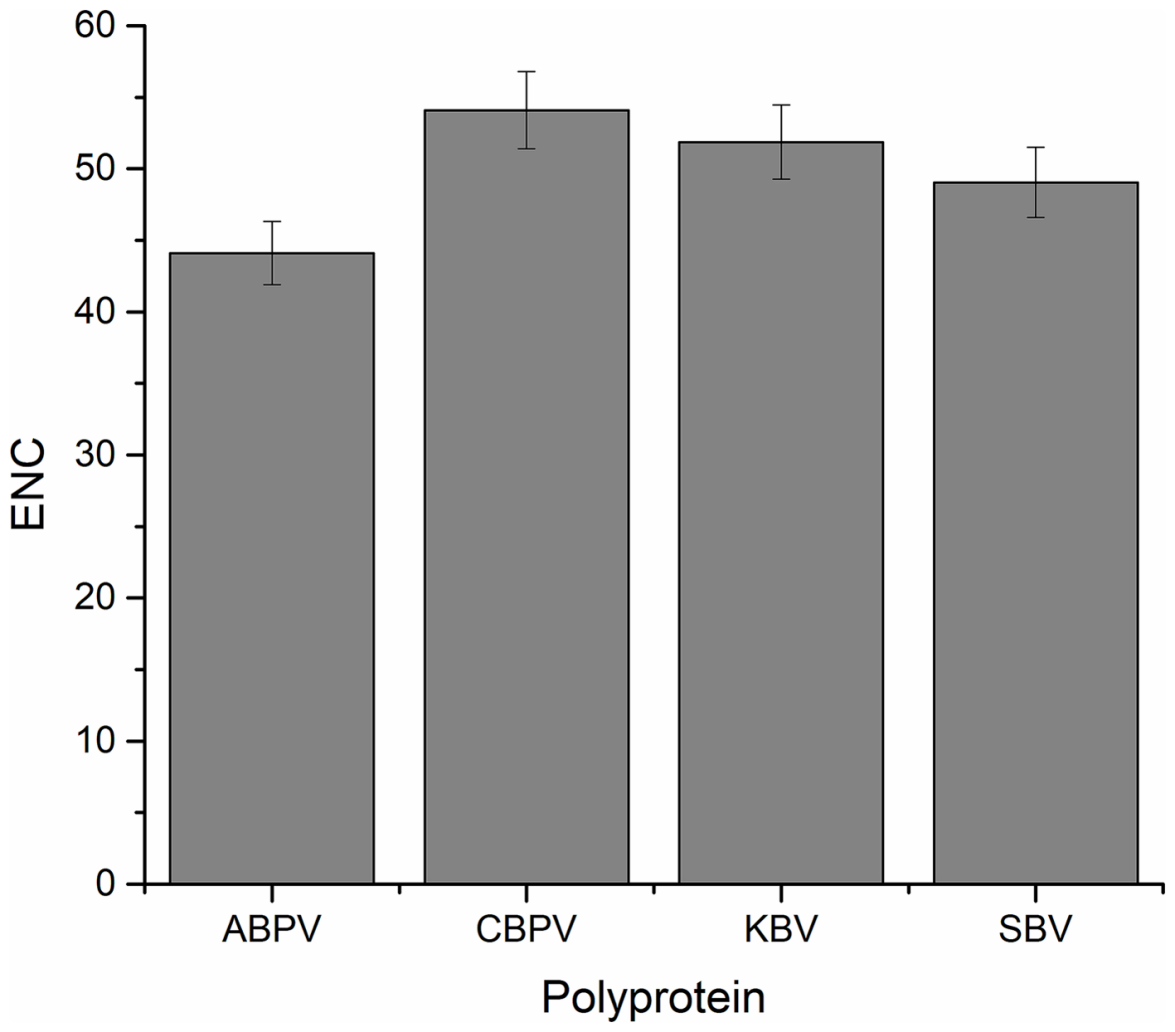
The effective number of codons (ENC) values for the polyprotein coding sequences of four HVs.

The CUB of polyprotein coding sequences in the four HVs was assessed using the effective number of codons (ENC). Generally, a lower ENC value indicates a stronger preference for certain codons. The average ENC values for the polyprotein coding sequences of ABPV, CBPV, KBV, and SBV were 44.10, 54.10, 51.86, and 49.05, respectively (Figure 1). These values indicate a conserved genomic structure and stable with minimal codon usage bias in all the polyprotein coding sequences analyzed.

### 3.3 Polyprotein coding sequences preferentially use A- and U-ending codons

We carried out an RSCU analysis to investigate the codon usage patterns in the polyprotein coding sequences of four HVs (Table 2). Out of the 18 preferred codons (RSCU > 1) common to all four HVs, 10 ended in U (UUU, GUU, AUU, UCU, UAU, GCU, CAU, AAU, UGU, and GAU), while 8 ended in A (UUA, CCA, CAA, ACA, AAA, AGA, GAA, and GGA). These results indicate that codons ending in A and U are favored in the polyprotein coding sequences of the four HVs. Almost all the favored and non-favored codons lie in the range from 0.6 to 1.6. Among the 59 codons, 3 were over-represented (RSCU > 1.6) in all the polyprotein coding sequences of the four HVs, namely UUA, AGA, and GGA. Conversely, 7 out of the 59 codons were under-represented (RSCU < 0.6), such as CUG, AGC, GCG, UAC, UCG, CGG, and GGG. Interestingly, the results also revealed that all overrepresented codons ended with A, while the most underrepresented codons ended with G, suggesting that mutational bias played a key role in influencing the codon usage patterns in polyprotein coding sequences.

**Table 2:** The relative synonymous codon usage frequency of polyprotein coding sequences in HVs and its hosts.

AA	Codons	RSCU (Mean)	*Apis mellifera*	*Apis cerana*	*Vespa velutina*	*Varroa destructor*
Phe (F)	**UUU**	**1.25**	1.58	1.44	1.39	1.29
	UUC	0.75	0.42	0.56	0.61	0.71
Leu (L)	**UUA**	**1.62**	3.07	2.51	1.83	1.40
	UUG	1.52	1.03	1.10	0.90	1.16
	CUU	0.94	0.78	0.96	1.20	1.07
	CUC	0.94	0.25	0.52	1.09	0.63
	CUA	0.61	0.60	0.56	0.61	0.92
	CUG	0.38	0.27	0.35	0.37	0.82
Ile (I)	**AUU**	**1.23**	1.28	1.36	1.12	1.15
	AUC	0.74	0.33	0.45	0.55	0.69
	AUA	1.03	1.38	1.19	1.33	1.16
Val (V)	**GUU**	**1.46**	1.60	1.48	1.31	1.29
	GUC	0.69	0.41	0.57	0.70	0.78
	GUA	1.12	1.49	1.22	1.25	1.11
	GUG	0.74	0.50	0.72	0.75	0.82
Ser (S)	**UCU**	**1.54**	1.57	1.48	2.05	1.17
	UCC	0.95	0.47	0.75	0.70	0.68
	**UCA**	**1.46**	1.94	1.42	0.99	1.16
	UCG	0.48	0.58	1.06	1.08	0.90
	AGU	0.99	1.00	0.84	0.75	1.08
	AGC	0.58	0.44	0.45	0.44	1.02
Pro (P)	CCU	1.26	1.12	1.18	1.37	1.21
	CCC	0.60	0.40	0.69	0.75	0.69
	**CCA**	**1.54**	1.92	1.36	1.14	1.25
	CCG	0.60	0.56	0.77	0.74	0.86
Thr (T)	ACU	1.20	1.21	1.03	0.93	1.07
	ACC	0.73	0.33	0.51	0.56	0.71
	**ACA**	**1.42**	1.98	1.54	1.42	1.33
	ACG	0.65	0.48	0.92	1.10	0.90
Ala (A)	**GCU**	**1.49**	1.10	1.02	1.04	1.23
	GCC	0.75	0.43	0.65	0.63	0.80
	GCA	1.26	1.87	1.38	1.34	1.21
	GCG	0.50	0.59	0.95	0.99	0.76
Tyr (Y)	**UAU**	**1.44**	1.71	1.60	1.49	1.24
	UAC	0.56	0.29	0.40	0.51	0.76
His (H)	**CAU**	**1.22**	1.58	1.36	1.27	1.18
	CAC	0.78	0.42	0.64	0.73	0.82
Gln (Q)	**CAA**	**1.28**	1.60	1.52	1.42	1.16
	CAG	0.72	0.40	0.48	0.58	0.84
Asn (N)	**AAU**	**1.26**	1.67	1.58	1.40	1.16
	AAC	0.74	0.33	0.42	0.60	0.84
Lys (K)	**AAA**	**1.16**	1.70	1.60	1.47	1.32
	AAG	0.84	0.30	0.40	0.53	0.68
Asp (D)	**GAU**	**1.26**	1.63	1.53	1.36	1.15
	GAC	0.74	0.37	0.47	0.64	0.85
Glu (E)	**GAA**	**1.26**	1.67	1.48	1.14	1.30
	GAG	0.74	0.33	0.52	0.86	0.70
Cys (C)	**UGU**	**1.16**	1.44	1.33	1.34	1.14
	UGC	0.84	0.56	0.67	0.66	0.86
Arg (R)	CGU	0.96	0.69	0.98	1.00	1.09
	CGC	0.93	0.34	0.56	0.48	0.78
	CGA	1.06	1.10	1.41	1.17	1.11
	CGG	0.47	0.24	0.44	0.36	0.66
	**AGA**	**1.72**	3.08	1.96	2.32	1.44
	AGG	0.86	0.54	0.64	0.67	0.92
Gly (G)	GGU	0.97	1.20	0.93	1.03	1.11
	GGC	0.71	0.62	0.63	0.61	1.07
	**GGA**	**1.76**	1.79	1.74	1.62	1.13
	GGG	0.56	0.39	0.70	0.74	0.70

The most preferred codon used in coding each amino acid is shown in bold. Blue and green color marked the over-represented (RSCU>1.6) and under-represented (RSCU<0.6) codons, respectively. Codons with underline represent the preferred codons for both HVs and their host.

To assess if the CUB of the polyprotein coding sequences in the four HVs is affected by their hosts, a comparison was made between the codon usage patterns of the polyprotein coding sequences and those of the host organisms, including *Apis mellifera*, *Apis cerana*, *Vespa velutina*, and *Varroa destructor*. The results revealed that, for *Apis mellifera*, 37 out of 59 synonymous codons were selected in a similar manner; for *Apis cerana* and *Vespa velutina*, 43 out of 59 synonymous codons were selected identically; and for *Varroa destructor*, 47 out of 59 synonymous codons were selected in the same way. Interestingly, certain codons, including UUU, UUA, AUU, GUU, and UCU, showed similarities between the polyprotein coding sequences and their hosts, suggesting a potential link to virulence in the host species. On the other hand, codons such as UCG, GCG, CAG, AAC, and CGC displayed substantial differences between the polyprotein coding sequences and their hosts. RSCU analysis suggested that compositional constraints, specifically A and U, have largely influenced the preferred codons.

### 3.4 Impact of relative dinucleotide abundance on codon usage bias

**Figure 2:**
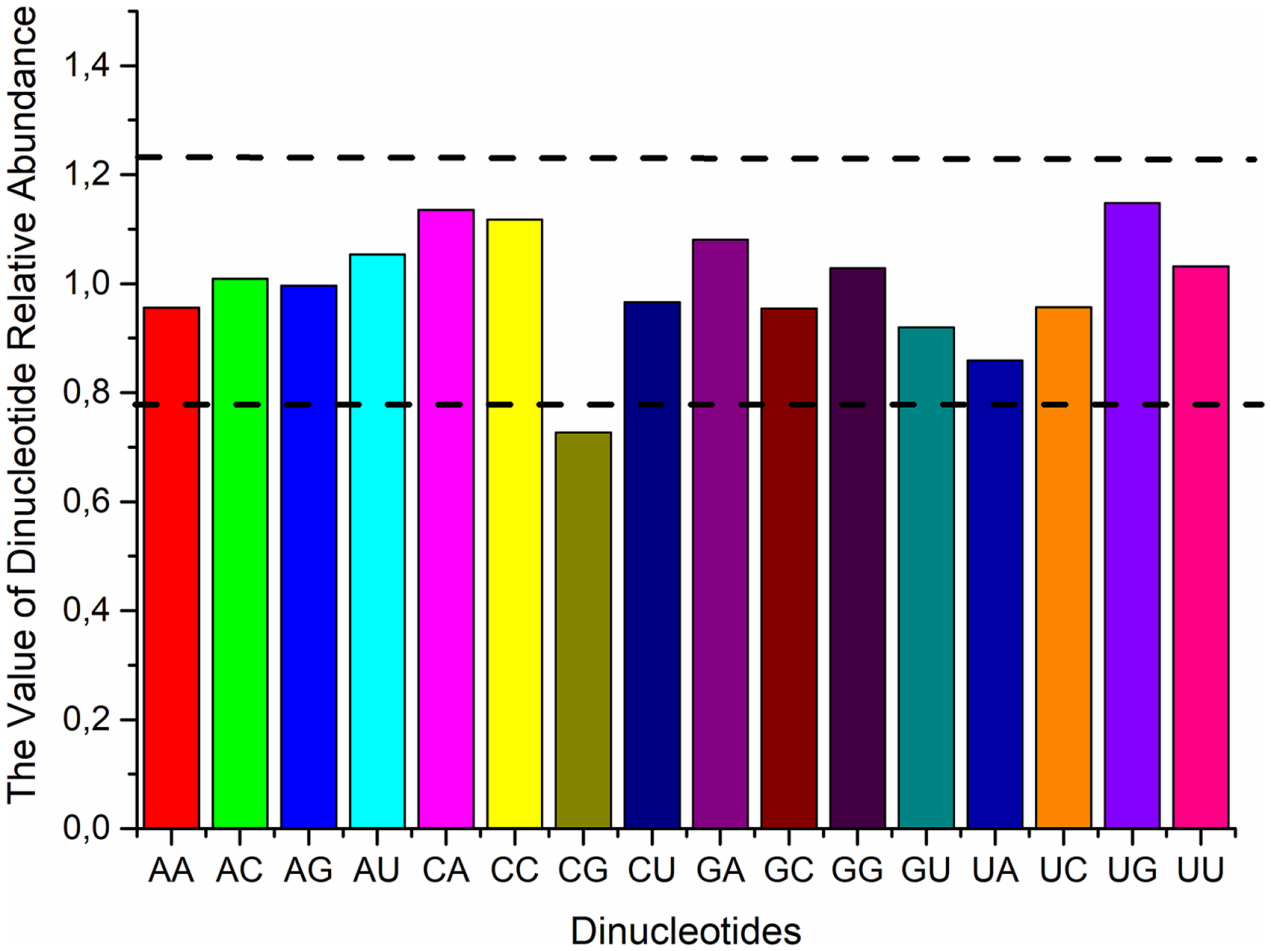
The average relative abundance of 16 dinucleotides in the polyprotein coding sequences of four HVs. Dashed lines indicate overrepresentation (Pxy > 1.23) or underrepresentation (Pxy < 0.78) of dinucleotides.

The frequency of dinucleotides impacts codon usage (46). The relative abundances of the 16 dinucleotides in the polyprotein coding sequences of four HVs were ascertained in order to look into the possible influence of dinucleotides on codon usage. As shown in Figure 2, we found that the polyprotein coding sequences did not have uniform distributions of the frequencies of occurrence of each dinucleotide. Specifically, the majority of dinucleotides (AC, AG, AU, GA, GG, and UU) were consistent with the theoretical value, being close to 1, with means of 1.01, 1.00, 1.05, 1.08, 1.03, and 1.03, respectively. Additionally, the results revealed that none of the dinucleotides were over-represented, while CG was under-represented. Moreover, the RSCU values of the three codons containing CG (UCG, GCG, and CGG) indicate that these codons are not favored. In conclusion, besides the total base composition, the dinucleotide composition and CG suppression were linked to the variation in synonymous codon usage, suggesting that mutational pressure may play a role in limiting codon usage patterns within polyprotein coding sequences.

### 3.5 Factors influencing codon usage bias in polyprotein coding sequences

**Figure 3:**
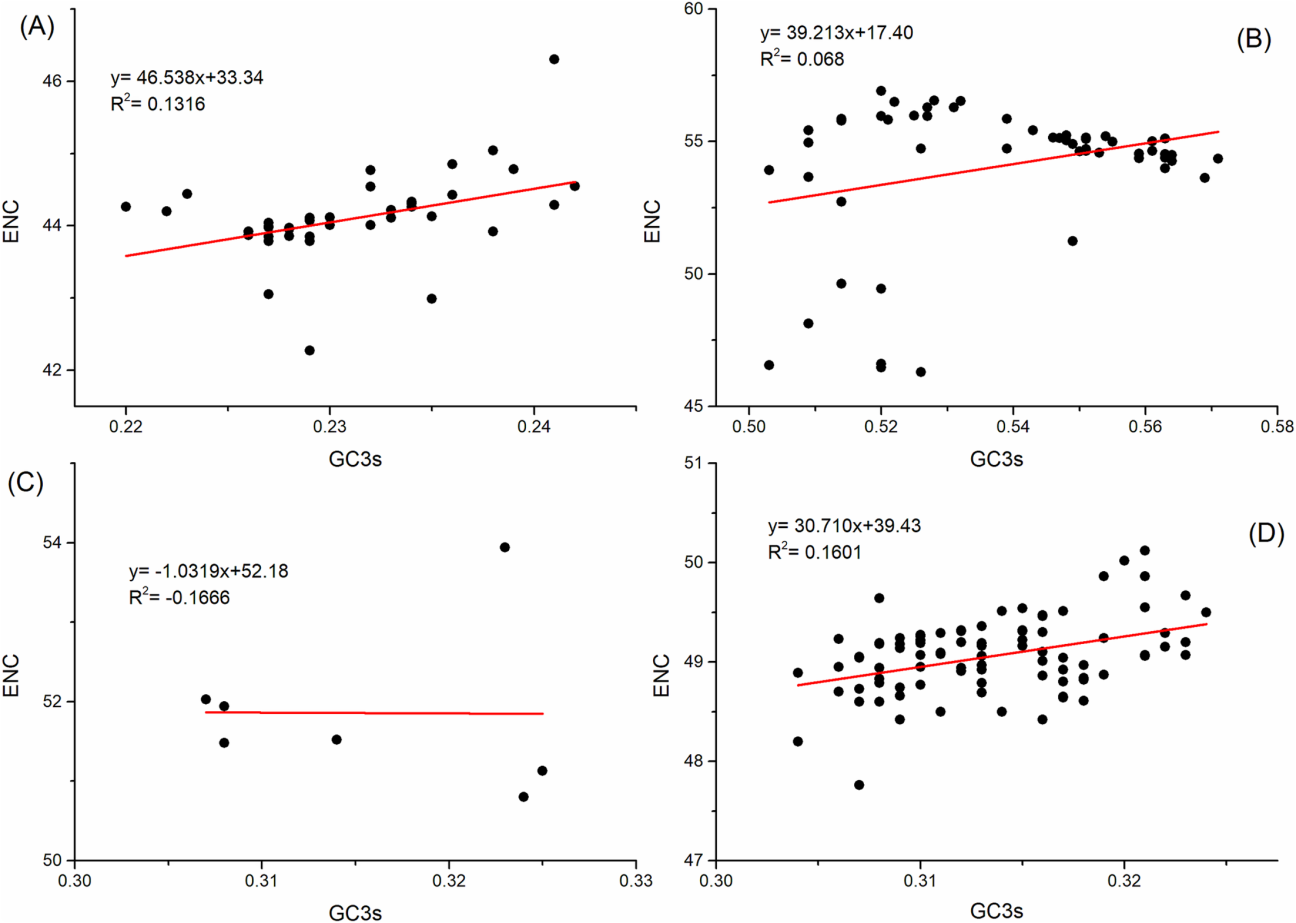
ENC-plot analysis of the polyprotein coding sequences for ABPV **(A)**, CBPV **(B)**, KBV **(C)**, and SBV **(D)**. ENC values are plotted against the GC3s content, with the solid line representing the expected ENC values for random codon usage based on GC3s.

To investigate the factors affecting codon usage patterns, ENC plot analysis, PR2 bias analysis, and neutrality analysis were applied to the polyprotein coding sequences. In the ENC plot analysis, some points are close to the theoretical fitting curve, while others fall below or on the curve, suggesting that both natural selection and mutation pressure contribute to the codon usage pattern of the polyprotein coding sequences of four HVs (Figure 3).

**Figure 4:**
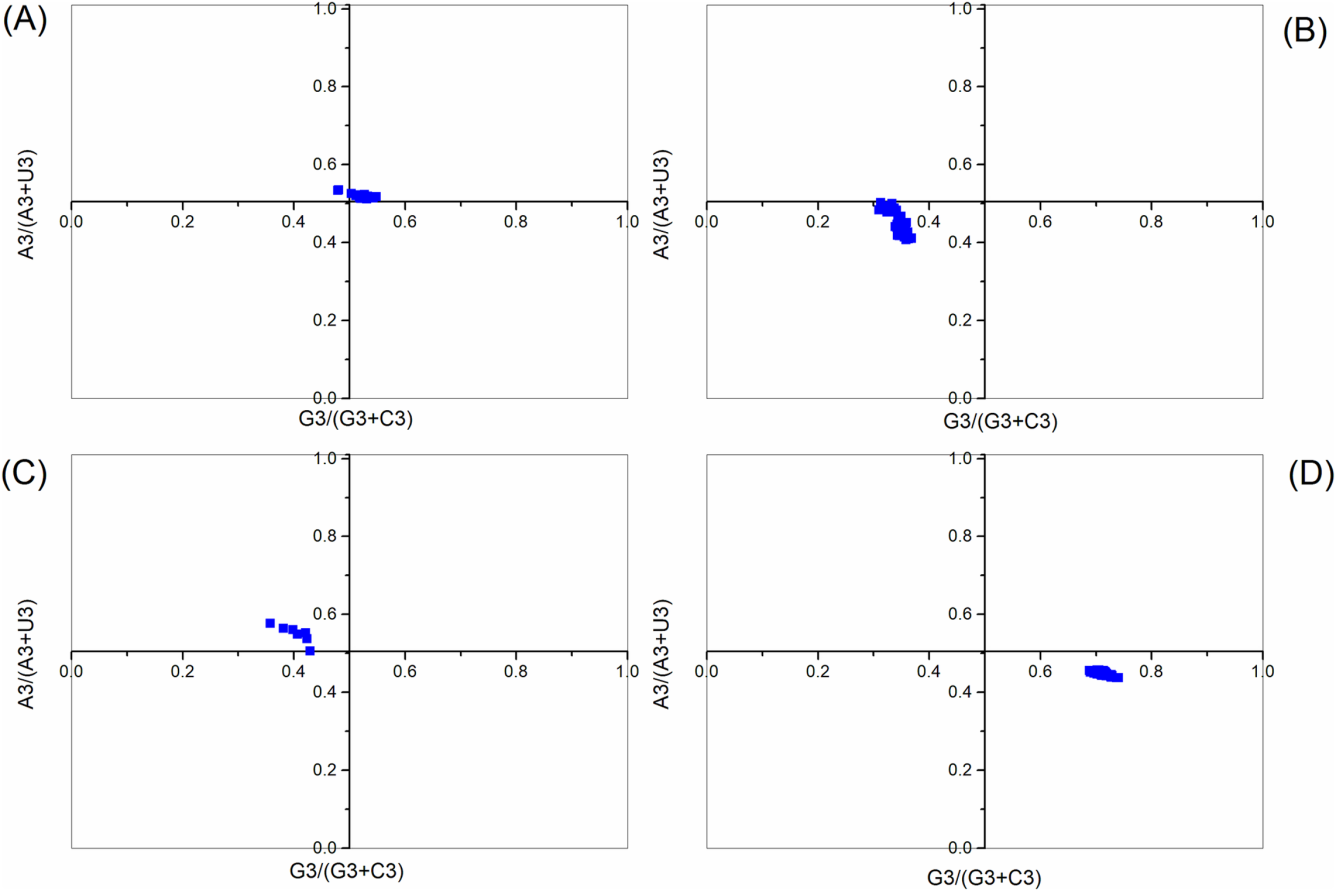
Parity rule 2 (PR2) plot analysis of the polyprotein coding sequences for ABPV **(A)**, CBPV **(B)**, KBV **(C)**, and SBV **(D)**. The center of the plot (coordinates at 0.5, 0.5) represents a position with no bias.

In the PR2 analysis, no bias in mutation pressure or selection is present when the plot is centered, with both coordinates at 0.5. The PR2 bias analysis showed that all points are distant from (0.5, 0.5), with the majority of points from the ABPV polyprotein coding sequences falling in the region where A3s/ (A3s + U3s) > 0.5 and G3s/ (G3s + C3s) > 0.5. This indicates a preference for A over U and G over C in the third codon position (Figure 4). Meanwhile, the CBPV polyprotein coding sequences were located in the region where A3s/ (A3s + U3s) < 0.5 and G3s/(G3s + C3s) < 0.5, suggesting that U is preferred over A and C is preferred over G in the third codon position. While the KBV polyprotein coding sequences fell in the region where A3s/ (A3s + U3s) > 0.5 and G3s/ (G3s + C3s) < 0.5, the SBV polyprotein coding sequences were in the region where A3s/ (A3s + U3s) < 0.5 and G3s/ (G3s + C3s) > 0.5. Thus, for KBV polyprotein coding sequences, A is favored over U, and C is favored over G at the third codon position, while for SBV polyprotein coding sequences, U is favored over A, and G is favored over C in the third codon position. These results demonstrate that mutational pressure, combined with factors like natural selection, influences the CUB of polyprotein coding sequences.

**Figure 5:**
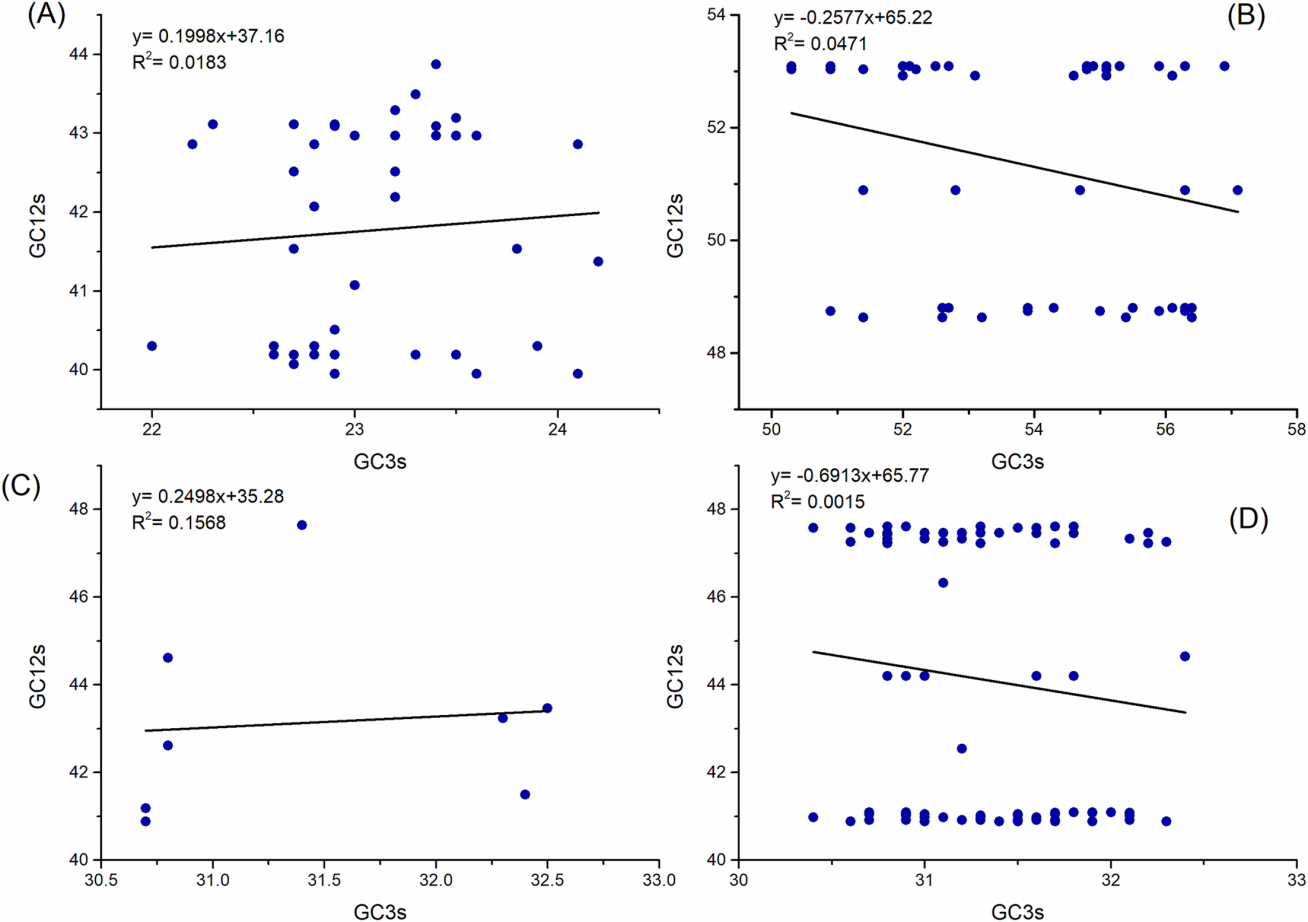
Neutrality analysis of the polyprotein coding sequences for ABPV **(A)**, CBPV **(B)**, KBV **(C)**, and SBV **(D)**, showing the relationship between GC12s and GC3s. The solid line represents the regression analysis of GC12s versus GC3s.

A neutrality plot can demonstrate the influence of natural selection and mutation pressure on the nucleotide content of genes. In the neutrality analysis, notable correlations were identified between the indices (*R*^2^ = 0.0183, *R*^2^ = 0.0471, *R*^2^ = 0.1568, and *R*^2^ = 0.0015; *p* < 0.05) for the polyprotein coding sequences of ABPV, CBPV, KBV, and SBV, respectively (Figure 5). The slopes of the regression lines for these polyprotein coding sequences were determined to be 0.1998, 0.2577, 0.2498, and 0.6913, respectively. This indicates that mutation pressure accounted for 19.98, 25.77, 24.98, and 69.13%, respectively, while natural selection contributed 80.02, 74.23, 75.02, and 30.87% to the CUB in the polyprotein coding sequences of ABPV, CBPV, KBV, and SBV, respectively. In summary, natural selection has a more substantial impact than mutation pressure in determining the codon usage patterns of the polyprotein coding sequences of ABPV, CBPV, and KBV. However, mutation pressure is the dominant factor influencing the codon usage bias in the polyprotein coding sequences of SBV.

### 3.6 Codon usage adaptation in polyprotein coding sequences

The codon adaptation index (CAI) was used to examine how the virus adjusts its codon usage to better adapt to the host. CAI values were calculated by comparing the codon usage of the polyprotein coding sequences to the codon usage of their hosts (*Apis mellifera*, *Apis cerana*, *Vespa velutina*, and Var*roa destructor*), which served as reference sets (Table 3). Higher CAI values, nearing 1, indicate a stronger adaptation to the host, whereas lower values, closer to 0, reflect weaker adaptation. The mean CAI values for the ABPV polyprotein coding sequences were 0.83, 0.88, 0.89 and 0.67 for the *Apis cerana*, *Apis mellifera*, *Varroa destructor*, and *Vespa velutina*, respectively. In comparison, the mean CAI values for the CBPV polyprotein coding sequences were 0.64, 0.74, and 0.87 for the *Apis cerana*, *Apis mellifera*, *Varroa destructor*, respectively. The mean CAI value for the KBV polyprotein coding sequences was 0.82 for the *Apis mellifera*, while the mean CAI values for the SBV polyprotein coding sequences were 0.78 and 0.84 for the *Apis cerana*, *Apis mellifera*, respectively. These values suggest that the polyprotein coding sequences exhibited a high level of host adaptation for all the hosts.

**Table 3:** The CAI values and the RCDI values of polyprotein coding sequences in HVs for corresponding hosts.

Virus	Host	CAI	RCDI
ABPV	*Apis cerana*	0.83	1.18
	*Apis mellifera*	0.88	1.16
	*Varroa destructor*	0.89	1.13
	*Vespa velutina*	0.67	1.62
CBPV	*Apis cerana*	0.64	1.62
	*Apis mellifera*	0.74	1.45
	*Varroa destructor*	0.87	1.11
KBV	*Apis mellifera*	0.82	1.18
SBV	*Apis cerana*	0.78	1.11
	*Apis mellifera*	0.84	1.14

In addition, we conducted a relative codon deoptimization index (RCDI) analysis to evaluate the combined impact of codon biases on gene expression (Table 3). The mean RCDI values for the ABPV polyprotein coding sequences were highest in *Vespa velutina*, followed by *Apis mellifera*, Var*roa destructor*, and *Apis cerana*. For the CBPV polyprotein coding sequences, the highest mean RCDI values were observed in *Apis cerana*, followed by *Apis mellifera* and *Varroa destructor*. In the case of the SBV polyprotein coding sequences, the highest mean RCDI values were found in *Apis mellifera*, followed by *Apis cerana*. The findings indicated that codon usage deoptimization of ABPV, CBPV, and SBV was the highest for *Vespa velutina*, *Apis cerana* and *Apis mellifera*, respectively. The results showed that codon usage deoptimization was highest for *Vespa velutina* in ABPV, for *Apis cerana* in CBPV, and for *Apis mellifera* in SBV.

### 3.7 Correspondence analysis of codon bias in polyprotein coding sequences

**Figure 6:**
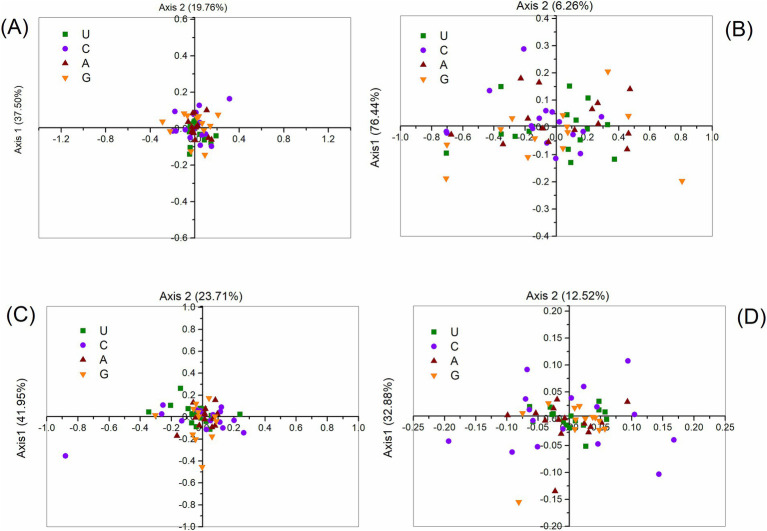
Correspondence analysis of synonymous codon usage in the polyprotein coding sequences of ABPV **(A)**, CBPV **(B)**, KBV **(C)**, and SBV **(D)**. The analysis utilizes the RSCU values for the 59 synonymous codons.

COA was conducted to evaluate variations in synonymous codon usage based on the RSCU values within the polyprotein coding sequences of four HVs (ABPV, CBPV, KBV, and SBV). For ABPV, the first two axes accounted for 37.50 and 19.76% of the variation, respectively, while for CBPV, these values were 76.44 and 6.26%. Similarly, the first two axes for KBV explained 41.95 and 23.71% of the variation, and for SBV, they accounted for 32.88 and 12.52%, respectively. This indicates that codon usage bias is primarily driven by the values of the first and second axes. When codons were ranked based on RSCU values along these two primary axes, a clear separation was observed: A- and U-ending codons were distributed along axis 1, while C- and G-ending codons were dispersed along axis 2 (Figure 6). This pattern highlights the distinct distribution of codons with different base endings across the two axes.

### 3.8 Correlation analysis between CUB indices

The analysis of CUB in polyprotein coding sequences revealed significant correlations between various indices (Table 4). Specifically, while GC3s, C3s, and G3s demonstrated a high negative correlation with CAI, U3s and A3s demonstrated a robust positive correlation. The results suggest that codon usage in polyprotein coding sequences is associated with gene expression. The selection of specific codons in the polyprotein coding sequences is not arbitrary; instead, it appears to be related to the level of gene expression. A3s and U3s show a positive correlation with CAI, which implies that A/U-rich codons are favored in genes with high expression, while G3s and C3s exhibit a negative correlation, indicating that G/C-rich codons are linked to lower expression levels. This implies that there is selective pressure for A/U-rich codons to enhance translation efficiency in these polyprotein coding sequences. Moreover, we performed a correlation analysis between ENC and CAI, which revealed no significant correlation between the two factors in the polyprotein coding sequences.

**Table 4:** Correlation analysis of codon usage indices in the polyprotein coding sequences of HVs.

Indices	CAI	GC	GC12	Axis 1	Axis 2
U3s	0,94,658*	0	0	0	0
C3s	−0,98,178*	0	0	0	0
A3s	0,95,235*	0	0	0	0
G3s	−0,94,617*	0	0	0	0
GC3s	−0,96,622*	0,99,995*	0,99,799*	0	0
GC12	0	0,99,738*	1	0	0
ENC	0	0	0	0	0
GRAVY	−0,99,502*	0,97,389*	0,95,694*	0	0
AROMA	0,982*	0	0	0	0

*p value < 0.05.

Additionally, correlation analysis was conducted to assess the relationship between codon usage bias and the GRAVY and AROMA scores (Table 4). GRAVY exhibited a significant positive correlation with GC3s, GC12, and GC, but a significant negative correlation with A and CAI. However, AROMA is only positively correlated with CAI. Neither AROMA nor GRAVY show any correlation with Axis 1 and Axis 2. It was found that the aromaticity and hydrophobicity of the polyprotein coding sequences were weakly associated with the CUB, highlighting the role of natural selection in shaping the codon usage pattern of these sequences.

## 4 Discussion

Viruses pose a serious threat to the health and survival of honeybees (6, 47). Gaining insight into the evolution and host adaptation mechanisms of viruses that infect honeybees is essential for devising effective and environmentally friendly strategies to manage these diseases. Thus, examining CUB is crucial for investigating genetic evolution and comprehending the characteristics of gene expression (48). CUB is commonly observed across viruses, bacteria, animals, fungi, and plants (33, 49–52). The ‘mutation-selection drift’ theory has been utilized to describe the origin of CUB in genes (53, 54). This theory suggests that evolutionary factors, including the mutation pressure, selection of compositional constraints, and genetic drift within a population, may significantly influence CUB (55). To date, the codon usage patterns of the polyprotein coding sequences in four HVs (ABPV, CBPV, KBV, and SBV) have not been thoroughly explored. In this research, we conducted a systematic analysis of the codon usage patterns and the factors influencing CUB in the polyprotein coding sequences of these four viruses.

Nucleotide composition is a key factor in shaping codon usage in both genes and genomes. The nucleotide content analysis demonstrated that A was the most prevalent in the polyprotein coding sequences of the four HVs. Additionally, the third codon position of these sequences was rich in A/U and poor in G/C, a pattern that aligns with findings in black queen cell virus (BQCV) (29), deformed wing virus (DWV) (29), Israeli acute paralysis virus (IAPV) (29), transmissible gastroenteritis virus (TGEV) (56), duck hepatitis virus 1 (DHV-1) (57), and invertebrate iridescent virus 6 (IIV6) (58). The significant bias toward A and U nucleotides, along with a strong preference for A- and U-ending codons in the polyprotein coding sequences of the four HVs, led us to evaluate the overall CUB in these sequences using ENC analysis. The analysis showed a slight bias and relatively stable codon usage in the polyprotein coding sequences. Our findings align with those of RNA viruses, which typically exhibit a low CUB (29, 59, 60). Earlier studies proposed that the use of a diverse set of codons to encode amino acids (low CUB) and reduced gene expression in RNA viruses may minimize competition for the translation mechanism between the host and the virus, thereby enhancing the virus’s replication rate in the host genome (61, 62).

RSCU is a crucial metric for analyzing the codon usage bias of species. Based on RSCU analysis, A/U-ended codons were the most preferred and over-represented in the polyprotein coding sequences of the four HVs, while G/C-ended codons were the most under-represented. Organisms with AT-rich genomes, such as *Taenia saginata*, *Hemerocallis citrina*, and *Polygonatum* species, typically favor A or T in the third position of their coding sequences (63–65). In contrast, GC-rich species, like bacteria and fungi, tend to prefer G or C in the same position (66, 67). As a result, the unique compositional restrictions (specifically A and U) may be responsible for the CUB observed in the polyprotein coding sequences of these four HVs. This result aligns with previous study (29, 30, 68) but contrasts with another study (69), which found that C3s and G3s were more commonly used than U3s and A3s. Furthermore, we compared the host’s RSCU values with those of the polyprotein coding sequences. The results indicated that the codon usage pattern of the polyprotein coding sequences closely resembles that of the host. It has been noted that the virus can translate more effectively when the same synonymous codon is used (70).

Moreover, the analysis of relative dinucleotide abundance showed a distinct bias in the usage of dinucleotides within the four HVs polyprotein coding sequences, with a pronounced underrepresentation of CG dinucleotides. This observation is consistent with the findings that many RNA viruses decrease CG dinucleotides (56), which is thought to increase the stability and effectiveness of viral mRNA translation (71). The biased usage patterns of dinucleotides in HVs polyprotein coding sequences may play a role in its host adaptation and evolutionary dynamics mechanisms, highlighting the need for further research into the functional implications of these patterns on viral pathogenicity and fitness. In general, RNA viruses adjust to changes in their host and environment by modifying the structure of their genomes (72).

Codon usage bias, a crucial factor in virus evolution, is affected by multiple elements, such as mutational pressure, natural selection, the content of the genomic region, and the length of the gene (73). Earlier research on enterovirus A and Venezuelan equine encephalitis virus has proposed that the CUB in these viruses is influenced by natural selection, as evidenced by nucleotide content comparisons, ENC plot, PR2 plot and neutrality plot analyses (62, 74). To evaluate whether both mutational pressure and natural selection have impacted viral codon usage patterns, we conducted ENC plot and PR2 plot analyses. The findings suggested that mutational pressure may not be the sole factor influencing codon usage patterns, with natural selection also likely contributing to the codon usage patterns of the four HVs polyprotein coding sequences, in line with previous research (75, 76). Neutrality analysis was also conducted to evaluate the relative contributions of natural selection and mutational pressure in shaping the CUB. The findings revealed that natural selection was the dominant factor affecting the CUB in the four HVs polyprotein coding sequences, consistent with results from previous studies (34, 77). Furthermore, strong correlations were observed between GRAVY/AROMA and nucleotide content. AROMA and GRAVY exhibited a slight correlation with CAI, indicating that the properties of viral proteins also play a role in the variation of codon usage in HVs’ polyprotein coding sequences, highlighting the influence of natural selection in shaping the codon usage bias of these sequences.

In addition, COA analysis of RSCU showed that the first axis accounted for only part of the diversity in codon usage. Hence, we determined that natural selection, along with multiple other factors, probably influences the selective restrictions on codon bias in the polyprotein coding sequences of HVs. This finding is consistent with previous research (78, 79).

Host–parasite interactions have a major impact on the evolution and dynamics of infectious diseases (80). Various research have demonstrated that codon usage patterns are vital in virus-host interactions (81, 82). The CAI is commonly employed as a metric for gene expression and to evaluate how viral genes adapt to their hosts (83). In our study, CAI analysis revealed that the HVs polyprotein coding sequences demonstrated a high degree of host adaptation across all hosts. These four HVs are capable of replicating efficiently in multiple hosts. As a result, we suggest that these HVs likely maintain a dynamic balance between codon adaptation and codon deoptimization, enabling effective replication cycles across hosts with diverse codon usage patterns. The lack of a significant correlation between CAI and ENC in the HVs polyprotein coding sequences indicates that codon adaptation in these viruses is influenced by factors beyond mutational bias or natural selection for uniform codon usage (62, 84). In the present study, a positive correlation was observed between A3s and U3s and CAI, indicating a preference for A/U-rich codons in highly expressed genes (55, 85). Conversely, G3s and C3s showed a negative correlation with CAI, suggesting that G/C-rich codons are associated with lower gene expression levels (86, 87). These patterns imply that there is selective pressure favoring A/U-rich codons to enhance translational efficiency, particularly in polyprotein-coding genes. This observation aligns with previous findings in various viral genomes, where codon usage bias has been shown to reflect host-driven selection for efficient translation. For example, studies on RNA viruses have demonstrated a tendency toward A/U-rich codon usage in highly expressed genes, likely driven by host tRNA abundance and translational selection (88, 89).

In conclusion, this study effectively demonstrates that the intricate codon usage patterns in the polyprotein coding sequences of four HVs are influenced by a complex interaction of factors, including natural selection, mutation pressure, and nucleotide compositional constraints. The CUB in polyprotein coding sequences of HVs was found to be low, predominantly driven by natural selection. Additionally, factors like dinucleotide frequencies, aromaticity, and hydrophobicity also play a role in shaping the codon usage pattern. The codon usage patterns in the polyprotein coding sequences of HVs were also observed to be affected by their host. In summary, our study deepens the understanding of the evolution of HVs polyprotein coding sequences, host-virus interactions, and the molecular mechanisms that drive viral adaptation.

## Data Availability

The datasets presented in this study can be found in online repositories. The names of the repository/repositories and accession number(s) can be found in the article/Supplementary material.

## Footnotes

^1^ https://www.ncbi.nlm.nih.gov/genbank/

^2^ https://www.bioinformatics.nl/cgi-bin/emboss/compseq
