# Supplementary material for: Comprehensive analysis of the codon usage patterns in the polyprotein coding sequences of the honeybee viruses
Source: Front Vet Sci. 2025 Jul 4;12:1567209. doi: 10.3389/fvets.2025.1567209 (PMC12270891; doi:10.3389/fvets.2025.1567209)
Supplement: Supplementary file 1 [file Table_1.docx]

Supplementary Material

**Supplementary Table 1. Sequence information of polyprotein coding sequences in four honeybee viruses**

| **Virus** | **Accession number** | **Host** |
| --- | --- | --- |
| ABPV | ON648748.1 | *Apis mellifera* |
|  | ON648738.1 | *Apis mellifera* |
|  | ON648739.1 | *Apis mellifera* |
|  | OR912387.1 | *Apis mellifera* |
|  | MN565031.1 | *Vespa velutina* |
|  | MZ821781.1 | *Apis mellifera* |
|  | MZ821787.1 | *Varroa destructor* |
|  | OL803814.1 | *Apis mellifera* |
|  | OL803813.1 | *Apis mellifera* |
|  | OM744312.1 | *Apis mellifera* |
|  | MZ821784.1 | *Varroa destructor* |
|  | MZ821782.1 | *Apis mellifera* |
|  | OM744313.1 | *Apis mellifera* |
|  | MZ821786.1 | *Varroa destructor* |
|  | MZ821773.1 | *Apis mellifera* |
|  | MZ821780.1 | *Apis mellifera* |
|  | OM744314.1 | *Varroa destructor* |
|  | MZ821785.1 | *Varroa destructor* |
|  | MZ821779.1 | *Apis mellifera* |
|  | MZ821778.1 | *Apis mellifera* |
|  | MZ821775.1 | *Apis mellifera* |
|  | MZ821771.1 | *Apis cerana* |
|  | MZ821783.1 | *Varroa destructor* |
|  | MZ821776.1 | *Apis mellifera* |
|  | MZ821774.1 | *Apis mellifera* |
|  | OK491519.1 | *Apis mellifera* |
|  | MZ821772.1 | *Apis mellifera* |
|  | MZ821777.1 | *Apis mellifera* |
|  | AY053375.1 | *Apis mellifera* |
|  | AY053374.1 | *Apis mellifera* |
|  | AY053372.1 | *Apis mellifera* |
|  | AF486072.2 | *Apis mellifera* |
|  | AY053371.1 | *Apis mellifera* |
|  | AY053370.1 | *Apis mellifera* |
|  | AY053367.1 | *Apis mellifera* |
|  | AY053368.1 | *Apis mellifera* |
|  | AY053366.1 | *Apis mellifera* |
|  | AF486073.2 | *Apis mellifera* |
|  | NC_002548.1 | *Apis mellifera* |
|  | HM228893.1 | *Apis mellifera* |
|  | HM228890.1 | *Apis mellifera* |
|  | OP628240.1 | *Apis mellifera* |
|  | MN510868.1 | Apis mellifera |
| CBPV | MF175174.1 | *Apis mellifera* |
|  | MZ821978.1 | *Apis mellifera* |
|  | MZ821954.1 | *Apis cerana* |
|  | MZ821980.1 | *Apis mellifera* |
|  | OM744322.1 | *Apis mellifera* |
|  | OK491521.1 | *Apis mellifera* |
|  | MZ821958.1 | *Apis cerana* |
|  | MZ821998.1 | *Varroa destructor* |
|  | MZ821990.1 | *Apis mellifera* |
|  | MZ821984.1 | *Apis mellifera* |
|  | MZ821962.1 | *Apis mellifera* |
|  | MZ821966.1 | *Apis mellifera* |
|  | MZ821968.1 | *Apis mellifera* |
|  | MZ821964.1 | *Apis mellifera* |
|  | MZ821970.1 | *Apis mellifera* |
|  | MZ821996.1 | *Apis mellifera* |
|  | MZ821986.1 | *Apis mellifera* |
|  | MZ821956.1 | *Apis cerana* |
|  | MZ821994.1 | *Apis mellifera* |
|  | MZ821988.1 | *Apis mellifera* |
|  | MZ821976.1 | *Apis mellifera* |
|  | KU950354.1 | *Apis mellifera* |
|  | MZ821974.1 | *Apis mellifera* |
|  | MZ821972.1 | *Apis mellifera* |
|  | OM744324.1 | *Apis mellifera* |
|  | MZ821982.1 | *Apis mellifera* |
|  | MZ821960.1 | *Apis mellifera* |
|  | MZ821992.1 | *Apis mellifera* |
|  | KY937972.1 | *Apis mellifera* |
|  | ON648752.1 | *Apis mellifera* |
|  | ON648750.1 | *Apis mellifera* |
|  | MK637523.1 | *Apis mellifera* |
|  | NC_010712. | *Apis mellifera* |
|  | EU122230.1 | *Apis mellifera* |
|  | OR863169.1 | *Apis mellifera* |
|  | OR863175.1 | *Apis mellifera* |
|  | OR863173.1 | *Apis mellifera* |
|  | OR863171.1 | *Apis mellifera* |
|  | FJ345347.1 | *Apis mellifera* |
|  | FJ345348.1 | *Apis mellifera* |
|  | FJ345346.1 | *Apis mellifera* |
|  | FJ345339.1 | *Apis mellifera* |
|  | FJ345328.1 | *Apis mellifera* |
|  | FJ345340.1 | *Apis mellifera* |
|  | FJ345329.1 | *Apis mellifera* |
|  | FJ345349.1 | *Apis mellifera* |
|  | FJ345334.1 | *Apis mellifera* |
|  | FJ345330.1 | *Apis mellifera* |
|  | FJ345341.1 | *Apis mellifera* |
|  | FJ345332.1 | *Apis mellifera* |
|  | FJ345345.1 | *Apis mellifera* |
|  | FJ345344.1 | *Apis mellifera* |
|  | FJ345336.1 | *Apis mellifera* |
|  | FJ345335.1 | *Apis mellifera* |
|  | FJ345337.1 | *Apis mellifera* |
|  | FJ345333.1 | *Apis mellifera* |
|  | FJ345338.1 | *Apis mellifera* |
| KBV | NC_004807.1 | *Apis mellifera* |
|  | HM228887.1 | *Apis mellifera* |
|  | HM228885.1 | *Apis mellifera* |
|  | MT096516.1 | *Apis mellifera* |
|  | MW314660.1 | *Apis mellifera* |
|  | KF956377.1 | *Apis mellifera* |
|  | MN296283.1 | *Apis mellifera* |
|  | AY275710.1 | *Apis mellifera* |
| SBV | KX663835.1 | *Apis cerana* |
|  | JX270800.1 | *Apis cerana* |
|  | JX270795.1 | *Apis cerana* |
|  | JX270796.1 | *Apis cerana* |
|  | JX270797.1 | *Apis cerana* |
|  | JX194121.1 | *Apis cerana* |
|  | HM237361.1 | *Apis mellifera* |
|  | KX668140.1 | *Apis cerana* |
|  | MZ821922.1 | *Apis cerana* |
|  | KX668141.1 | *Apis cerana* |
|  | MH107056.1 | *Apis cerana* |
|  | KM495267.1 | *Apis cerana* |
|  | KU574661.1 | *Apis cerana* |
|  | KP296802.1 | *Apis cerana* |
|  | HQ322114.1 | *Apis cerana* |
|  | KM884993.1 | *Apis cerana* |
|  | MZ821923.1 | *Apis cerana* |
|  | MZ821932.1 | *Apis mellifera* |
|  | MG733283.1 | *Apis mellifera* |
|  | MZ821920.1 | *Apis cerana* |
|  | MZ821931.1 | *Apis mellifera* |
|  | KJ959614.1 | *Apis cerana* |
|  | KM884990.1 | *Apis cerana* |
|  | KM884991.1 | *Apis cerana* |
|  | KM884992.1 | *Apis cerana* |
|  | OM744330.1 | *Apis cerana* |
|  | KP296803.1 | *Apis cerana* |
|  | MN395732.1 | *Apis cerana* |
|  | MH509439.1 | *Apis cerana* |
|  | MZ821926.1 | *Apis mellifera* |
|  | MK719542.1 | *Apis cerana* |
|  | KP296801.1 | *Apis mellifera* |
|  | KC285046.1 | *Apis cerana* |
|  | JQ390592.1 | *Apis mellifera* |
|  | MN266898.1 | *Apis cerana* |
|  | MZ821921.1 | *Apis cerana* |
|  | MZ821930.1 | *Apis mellifera* |
|  | MZ821927.1 | *Apis mellifera* |
|  | MZ821919.1 | *Apis cerana* |
|  | MN082652.1 | *Apis cerana* |
|  | MZ821937.1 | *Apis mellifera* |
|  | MZ821936.1 | *Apis mellifera* |
|  | MN266899.1 | *Apis cerana* |
|  | KM884994.1 | *Apis cerana* |
|  | MZ821933.1 | *Apis mellifera* |
|  | MZ821928.1 | *Apis mellifera* |
|  | MZ821934.1 | *Apis mellifera* |
|  | PP745030.1 | *Apis mellifera* |
|  | MZ821924.1 | *Apis mellifera* |
|  | MN528599.1 | *Apis cerana* |
|  | MZ821935.1 | *Apis mellifera* |
|  | KY465677.1 | *Apis mellifera* |
|  | KY465674.1 | *Apis mellifera* |
|  | NC_002066. | *Apis mellifera* |
|  | KY465672.1 | *Apis mellifera* |
|  | KY273489.1 | *Apis mellifera* |
|  | KY465671.1 | *Apis mellifera* |
|  | KY465675.1 | *Apis mellifera* |
|  | KY465673.1 | *Apis mellifera* |
|  | JX270798.1 | *Apis cerana* |
|  | JX270799.1 | *Apis cerana* |
|  | KC007374.1 | *Apis cerana* |
|  | KJ959613.1 | *Apis cerana* |
|  | KM884995.1 | *Apis mellifera* |
|  | KX668139.1 | *Apis cerana* |
|  | AF469603.1 | *Apis cerana* |
|  | KJ000692.1 | *Apis cerana* |
|  | KF960044.1 | *Apis cerana* |
|  | KU574662.1 | *Apis cerana* |
|  | MZ821929.1 | *Apis mellifera* |
|  | KY774627.1 | *Apis mellifera* |
|  | KX819276.1 | *Apis mellifera* |
|  | KY774628.1 | *Apis mellifera* |
|  | MZ821925.1 | *Apis mellifera* |
|  | MN082651.1 | *Apis mellifera* |
|  | KY465679.1 | *Apis mellifera* |
|  | KY465676.1 | *Apis mellifera* |
|  | KP296800.1 | *Apis mellifera* |
|  | JQ390591.1 | *Apis mellifera* |
|  | MT636329.1 | *Apis mellifera* |
|  | MT636331.1 | *Apis mellifera* |
|  | MT636334.1 | *Apis mellifera* |
|  | MT636328.1 | *Apis mellifera* |
|  | KY465678.1 | *Apis mellifera* |
|  | MT636333.1 | *Apis mellifera* |
|  | OR496426.1 | *Apis mellifera* |
|  | MT636336.1 | *Apis mellifera* |
|  | MT636327.1 | *Apis mellifera* |
|  | MT636332.1 | *Apis mellifera* |
|  | MT636330.1 | *Apis mellifera* |
|  | OL803870.1 | *Apis mellifera* |
|  | MT636335.1 | *Apis mellifera* |
|  | PQ376985.1 | *Apis mellifera* |
|  | OR496422.1 | *Apis mellifera* |
|  | KY887698.1 | *Apis mellifera* |
|  | MG545287.1 | *Apis mellifera* |
